# Acetylation improves thermal stability and transmittance in FOLED substrates based on nanocellulose films†

**DOI:** 10.1039/c7ra11134g

**Published:** 2018-01-17

**Authors:** Shuang Yang, Qiuxia Xie, Xiuyu Liu, Min Wu, Shuangfei Wang, Xueping Song

**Affiliations:** College of Light Industry and Food Engineering, Guangxi University Nanning 530004 PR China; Guangxi Key Laboratory of Clean Pulp & Papermaking and Pollution Control Nanning 530004 PR China

## Abstract

Bleached softwood pulp was used to prepare nanofibrillated cellulose (NFC) by mechanical grinding and a high-pressure homogenization process. Acetylation improved the aspect ratio and dispersion of the NFC; however, highly acetylated NFC was not able to form a film by vacuum filtration if the NFC : acetic anhydride (AA) ratio was greater than 1 : 6. An NFC film prepared by acetylated NFC has potential as a flexible organic light-emitting device (FOLED) substrate. Acetylation improved the thermal stability and transmittance of NFC films, which were optimal at 5.43 ppm K^−1^ and 65%, respectively, when the ratio of NFC : AA was 1 : 3. Moreover, both the mechanical properties and flexibility of the NFC films were well maintained when the NFC : AA ratio was 1 : 3. Additionally, all NFC films prepared by acetylated NFC were smooth, flat, and uniform.

## Abstract 2

Bleached softwood pulp was used to prepare nanofibrillated cellulose (NFC) by mechanical grinding and a high-pressure homogenization process.

## Graphical abstract

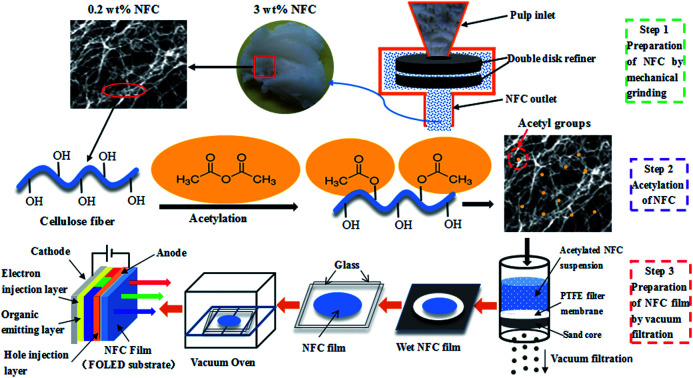

## Introduction

Optoelectronic technology and flexible electronic materials have attracted rapidly growing interest over the past 10 years in relation to the field of electronic displays. A flexible organic light-emitting device (FOLED) has some remarkable advantages, such as light weight, low power consumption, long equipment life, and outstanding flexibility.^1,2^ The FOLED substrate is a very important part of a flexible electronics display, which needs to provide hard mechanical support for equipment at the same time as facilitating photonic and electronic processes. The substrate performance will eventually determine the flexibility, portability, optical properties, and production methods of the FOLED.^3,4^ The performance of the FOLED substrate should be smooth, and should demonstrate high flexibility, good thermal stability, and excellent mechanical properties. At present, the materials required to prepare a FOLED substrate predominantly include metal foil, ultrathin glass, and plastics.^4^ However, existing flexible substrates have various disadvantages, including poor transparency, high cost, high coefficient of thermal expansion (CTE), and frangibility.^5,6^ Therefore, it is necessary to develop a new FOLED substrate that possesses low CTE, good endurance, and excellent flexibility in order to satisfy the demands of FOLED development. In the context of green composites, a new kind of biodegradable, soft, and transparent substrate nanocellulose film has attracted the attention of researchers, and some academics believe that nanocellulose composite materials have potential as flexible display substrates.^5,7^

The term ‘nanocellulose’ refers to cellulose having at least one dimension of up to 100 nanometers in size, and predominantly includes cellulose nanocrystals, nanocrystalline cellulose, and nanofibrillated cellulose.^8^ Nanocellulose has many excellent properties, such as greater chemical reactivity owing to multitudinous hydroxyls on the surface, higher mechanical performance, qualitative light (1.5 g cm^−3^ density), larger specific surface area (>50 m^2^ g^−1^), and lower CTE value (8 ppm K^−1^),^9^ compared with glass substrate. Nanocellulose has found application in many fields due to its unique properties, including as a paper additive, biodegradable film, barrier packaging material, enhancer of composite materials, conductive film, electronic substrate, and multi-functional magnetic material.^10–14^ Nanofibrillated cellulose (NFC), prepared by mechanical grinding and high-pressure homogenization treatment, is obtained by the mechanical separation of the original fiber bundle, which is a semi-crystalline polymer of cellulose chains, and substantially retains the performance of the natural plant fiber. The fibers of NFC are very flexible and soft, and can create a three-dimensional mesh structure connected by hydrogen bonds or entanglements after removal of moisture.^15^ However, the characteristics of strong hydrophilicity, weak compatibility with matrices, and easy recombination severely limit the applications of NFC. Thus, NFC requires appropriate modification to allow practical use.

Methods of modifying NFC mainly include surface grafting, use of a silane coupling agent, acetylation, and surfactant modification.^16^ Among these methods, acetylation is one of the most promising due to the fact that the chemicals used in the process are very common and not particularly expensive.^17,18^ The hydroxyl groups of the glucose groups on NFC are replaced by acetyl groups during the modification process, and the interaction forces between the fibers are reduced, enhancing the NFC hydrophobic and dispersion properties, as well as its compatibility with other polymers.^19^ Furthermore, acetylation is also used to improve the thermal degradation of the cellulose fibers and the optical properties of the nanocellulose composite films.^20^

FOLED substrates also require high transmittance for clear imaging, and good thermal stability in order to withstand wrinkles, deformation, oxidation coloring, or thermal decomposition during the processes of preparation and usage. However, NFC films prepared using mechanical techniques show lower transmittance, and are not smooth and uniform due to the easy recombination and poor dispersion of NFC. Therefore, acetylation plays an important role in improving the properties of NFC films as FOLED substrates.

Previous studies have found that NFC may be modified in 1 h at normal temperature using toluene as the solvent and acetic anhydride (AA) as the modifier, reducing the energy and time required for acetylation.^5^ In terms of NFCs prepared by different materials and preparation methods, the dosage of AA has a considerable influence on its substitution.^5^ Thus, the influence of different NFC : AA ratios on the acetylation and the properties of the resulting NFC films was the focus of this study.

**Fig. 1:**
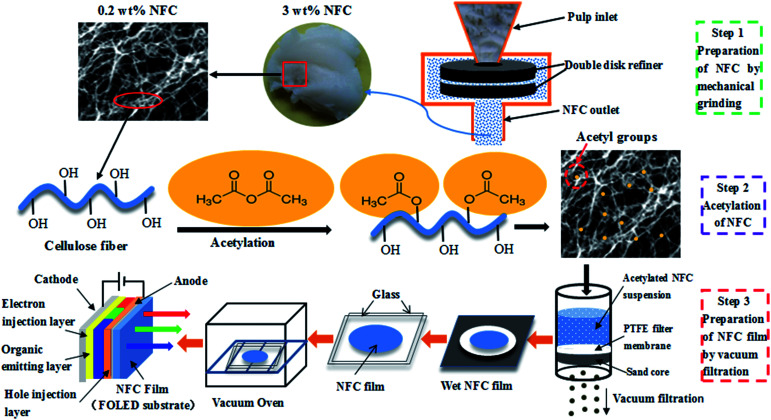
Preparation of NFC film as a FOLED substrate.

Several methods can be used to produce NFC films, including casting,^21^ spray coating,^22^ and vacuum filtration.^23^ Rapid preparation of NFC films with high surface smoothness and optical transparency is important to facilitate the development of FOLEDs. In this study, NFC was prepared by mechanical grinding and high-pressure homogenization treatment, and then was modified by acetylation. The NFC films were prepared using acetylated NFC by simulating the papermaking process, with reduced dewatering time.^24^ The method of preparing NFC films is sufficiently simple to readily produce FOLEDs with a roll-to-roll mode on an industrial scale. The preparation of NFC films as FOLED substrates is shown in Fig. 1. The objective of this study was to explore the feasibility of NFC films prepared from acetylated NFC as high-performance FOLED substrates by investigating their thermal properties, mechanical strength, and light transmittance.

## Experimental

### Materials

Bleached softwood kraft pulp (pinus khasys), provided by Yun-jiang Forestry & Pulp Mill Co., Ltd., China, was used as the starting material. The chemical composition of the pulp was 96.90% cellulose, 3.50% hemicellulose, and less than 0.1% lignin. All reagents, such as potassium hydroxide, sulfuric acid, sodium chlorite, acetic acid, AA, perchloric acid, ethanol, toluene, and acetone, were analytical reagent (AR) grade and were obtained from the Shanghai Aladdin Biochemical Technology Co., Ltd (Shanghai, China).

### Preparation of NFC

The 3 wt% pulp was ground at 1500 rpm for 30 min (Super Masscolloider MKZA 10-15JIV; Masuko Sangyo Co., Ltd., Saitama, Japan). After grinding, the pulp was diluted to 0.2 wt% pulp suspension and passed through a high-pressure homogenizer (GJJ-0.06/40; Keju fluid equipment manufacturing Co., Ltd., China). The conditions of homogenization were as follows: passing two times at 0 bars, passing three times at 400 bars, and passing three times at 600 bars.

### Acetylation of NFC

**Fig. 2:**
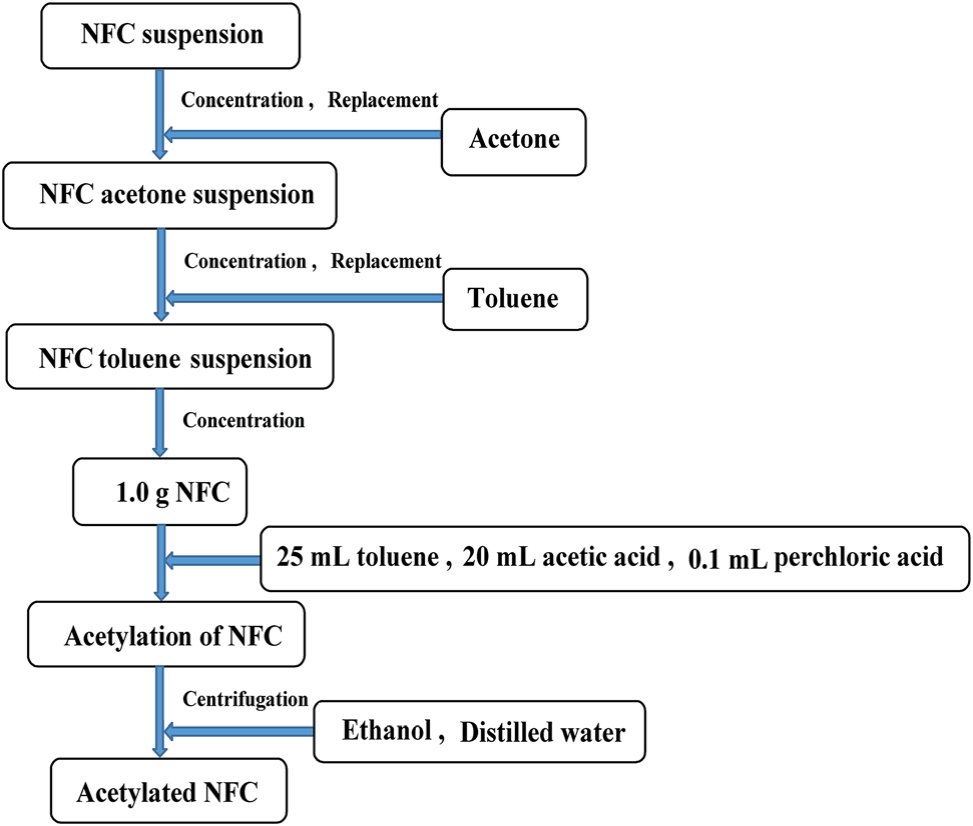
Preparation of acetylated NFC.

After homogenization, the NFC was acetylated. The preparation of acetylated NFC is shown in Fig. 2. The NFC suspension was replaced repeatedly with acetone through vacuum filtration in order to obtain an NFC acetone suspension. An NFC toluene suspension was obtained in a similar way. Then, NFC acetylation was conducted when 25 mL toluene, 20 mL acetic acid, and 0.1 mL perchloric acid were added to the NFC (83 wt%, 1.0 g bone dry) in sequence. AA was also added to the NFC in the necessary quantity (1, 2, 3, 4, 5, 6, 7, or 10 mL). The sample of NFC was stirred continuously, and the acetylation was maintained for 1 h at room temperature. After acetylation, the NFC was washed with ethanol and distilled water by centrifugal separation, respectively (repeated three times, 8 min per time, 10 000 rpm min^−1^). Finally, the acetylated NFC was obtained.

### Preparation of NFC films

The acetylated NFC slurry (0.3 g bone dry) was diluted to 0.2 wt%, and the suspension of diluted NFC was stirred for 2 h in order to ensure its dispersion. Then, the dispersed NFC was vacuum filtered using a G2 sand core funnel (90 mm diameter), which was padded with a layer of hydrophilic polytetrafluoroethylene organic filter membrane (0.22 μm pore size, 90 mm diameter) in advance. The wet NFC film was taken out together with the organic filter membrane after filtering, covering another organic filter membrane on the other side of the NFC film. The filter papers were covered on the surface of the organic filtering films before drying, speeding up the removal of moisture. The NFC film was pressed from both sides with glass in order to obtain a flat film during drying. Then the film was dried at room temperature for 12 h, and vacuum dried at 55 °C for 24 h. Finally, the dried and smooth NFC film was obtained.

### Analysis

#### Transmission electron microscopy (TEM)

The morphology and dimensions of NFC were observed using TEM (TECNAI G2 F30, US). The NFC suspension was diluted to 0.01 wt%, and dispersed for 30 min with ultrasound. A small amount of the NFC dispersion was carefully dropped onto a copper net coated with carbon, and allowed to stand for 5 min. Then the sample was dyed with a small amount of staining agent (3% phosphotungstic acid stain, pH 7.0). The excess liquid was removed with filter paper, and the sample was dried naturally. The particle size analysis of NFC was carried out using Nano Measurer 1.2.5 software. In addition, the NFC dimensions were obtained by measuring at least 100 individual fibers.

#### Determination of acetylation degree

The degree of substitution (DS) of acetylated NFC samples (DS < 3.0) was measured by ^1^H-nuclear magnetic resonance (NMR) spectroscopy (Bruker AC III HD600, Germany).^25^^1^H-NMR spectra were measured with a spectrometer using tetramethylsilane (TMS) as the internal standard, over 256 scanning times at 500 MHz. The sample of acetylated NFC was dissolved in dimethyl sulfoxide (DMSO)-d_6_, and the DS values were calculated according to the Goodlertt formula:^26^1
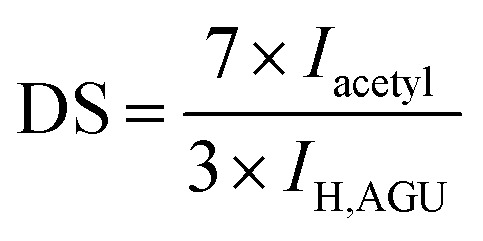
where *I*_acetyl_ is the peak integral of methyl protons of the acetyl moiety, *I*_H,AGU_ is the peak integral of all protons of the anhydroglucose unit (AGU), 7 is the number of protons on the AGU, and 3 is the number of protons on the methyl group.

#### Thermal performance

The samples, both before and after acetylation, were used to prepare NFC films. Thermal gravimetric analysis (TGA) of the NFC films was performed with a TGA-DSC/DTA analyzer (STA 449 F5, NETZSCH-Gerätebau GmbH, Germany). Each NFC film (10 mg) was heated from 30 to 600 °C with a heating rate of 10 °C min^−1^ in a nitrogen atmosphere. The flow of nitrogen was 20 mL min^−1^. Proteus analysis software was used to study the thermal stability of the samples. All experiments were carried out in triplicate, and the results were presented as average values.

CTE was used to characterize the thermal expansion performance of the NFC films prepared before and after acetylation of NFC. The CTE was measured using a thermomechanical analyzer (Q400, TA Instruments, US). The measurement conditions were as follows: specimen area 25 × 3 mm, pull 0.03 N, temperature from 30 to 150 °C with a heating rate of 5 °C min^−1^. The test was conducted under nitrogen conditions, and each sample was circulated three times. The CTE values were determined by the average value of the second run and the third run in order to eliminate the residual stress of the membrane material. CTE values were given as the average of three independent determinations for each sample.

#### Mechanical properties of NFC films

The Young's modulus, tensile strength, and elongation at breakage of the NFC films were measured using a Shimadzu AG-X testing machine (Kyoto, Japan). The specimen dimensions were 25 mm in length and 3 mm in width. The measurement conditions were as follows: load sensor 50 N, gage length 20 mm, and stretching rate 1 mm min^−1^. The Young's modulus, elongation at breakage, and tensile strength were obtained directly from the testing results. Three test samples were measured, and the data reported were an average of all tests.

#### Optical properties of NFC films

Using a single lens reflex (SLR) camera (Nikon d7100), the appearance of the NFC films was photographed in a well-lit laboratory. An ultraviolet-visible (UV-vis) spectrometer Lambda 950 (PerkinElmer, US) was used to measure the light transmittance of the NFC films in the visible wavelength range, from 380 to 780 nm. The samples were cut to 10 × 10 mm in size, and placed 25 cm from the outlet of the integral sphere.

## Results and discussion

### Effects of acetylation on morphology and dimensions of NFC

**Fig. 3:**
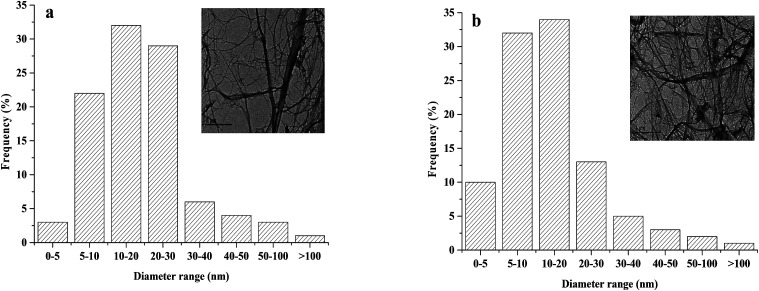
Size distribution for diameters and TEM images of fibers: (a) NFC (NFC : AA ratio of 1 : 0) and (b) acetylated NFC (NFC : AA ratio of 1 : 3).

By analyzing the size distribution of the diameters of the fibers modified by different NFC : AA ratios, we found that acetylation had little effect on fiber dimension. This observation was similar to the findings of Jonoobi *et al.*^27^ Thus, only images for NFC : AA ratios of 1 : 0 and 1 : 3 are exhibited herein. The fibers in the TEM images in Fig. 3 were very thin and presented obvious fine filaments, indicating that the NFC produced by mechanical grinding and high-pressure homogenization treatment had a large aspect ratio.

Moreover, a large aspect ratio could significantly improve the Young's modulus, the strength, and the transmittance values of the NFC film.^28^ The dimensions of NFC were obtained by measuring at least 100 individual fibers from the TEM micrographs, of which the diameters of 83% of individual NFC fibrils was estimated to be within the range of ∼5 to 30 nm. Moreover, the acetylated NFC had an average diameter of ∼5 to 20 nm. The smaller size might be due to the destruction of the fiber structure during NFC acetylation.

### Effects of acetylation on the DS of NFC

It is very important to control the DS of products during the modification process, as this will greatly affect the physicochemical properties of the product. ^1^H-NMR studies were carried out to elucidate the effects of AA on the DS of NFC and the mechanism of the acetyl-substituting hydroxyl group. Fig. S1† shows the effects of different NFC : AA ratios on the ^1^H-NMR spectra of NFC (see detail in the ESI†). The peak at 2.5 ppm was produced by the DMSO-d_6_ solvent.^29^ Related statistics show that the characteristic signals at *δ* ∼ 3.5–5.5 ppm were the peaks of all protons of AGU, and the peak at *δ* ∼ 1.8–2.1 ppm was due to the methyl protons of the acetyl moiety.^30^ Moreover, the peaks at 1.8 ppm, 1.9 ppm, and 2.1 ppm are assigned to the methyl hydrogen signals of the acetyl groups substituting the hydroxyl groups on C3, C2, and C6 of the glucose unit in cellulose, respectively.^31^ Therefore, the emergence of methyl hydrogen signals at ∼1.8 to 2.1 ppm indicates that NFC acetylation was successful. As shown in Fig. S1,† the weak absorption signal was produced at 1.9 ppm when the ratio of NFC : AA was 1 : 1 and 1 : 2 (see detail in the ESI†), which means that the C2 hydroxyl of the cellulose glucose unit had been replaced by acetyl. When the NFC : AA ratio increased from 1 : 3 to 1 : 6, besides the increase in absorption peak at 1.9 ppm, the absorption peak at 2.1 ppm appeared when the ratio of NFC : AA was 1 : 4 and was strengthened with the increase of NFC : AA ratio, which signified that the hydroxyl groups on C2 and C6 were replaced by acetyl groups, respectively. When the NFC : AA ratio was greater than 1 : 6, the increasing ratio of NFC : AA resulted in the enhancement of the absorption peak at 1.8, 1.9 and 2.1 ppm, which should be attributed to the replacement of C3, C2, and C6 hydroxyl groups with the acetyl groups. However, when the vast majority hydroxyl groups in the glucose units were replaced by acetyl groups, the hydrophobic group was increased dramatically in NFC, and the NFC degradation was aggravated due to the existence of more acid in the reaction system.^27^

**Fig. 4:**
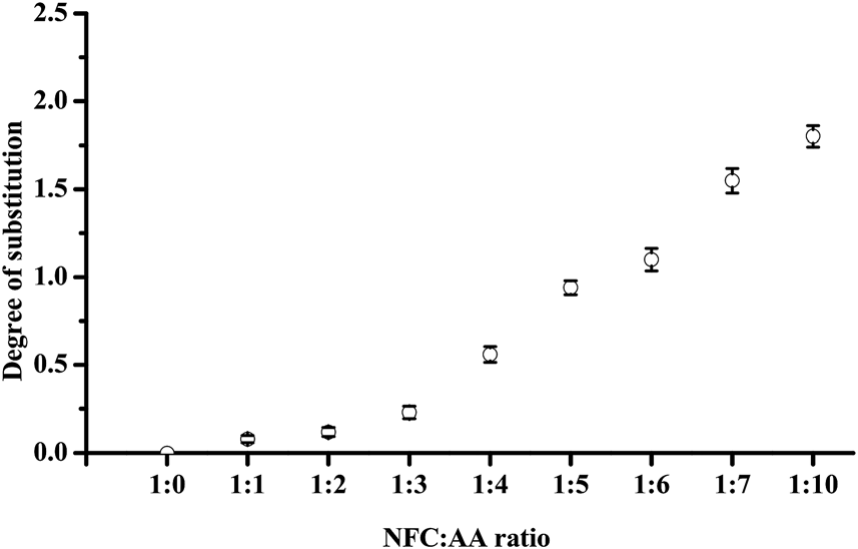
Effects of different NFC : AA ratios on the DS of NFC.

DS refers to the number of hydroxyl and AA reactions in each glucose unit of cellulose; with three hydroxyl groups in each of the glucose unit, the maximum theoretical value of DS is 3.^32^ The DS values of acetylated NFC were calculated using the Goodlertt formula. The results showed that the degree of acetylation was not uniform and was constantly enhanced with increasing NFC : AA ratio. Further, different NFC : AA modification ratios had a considerable influence on the hydroxyl substitution. When the ratio of NFC : AA was 1 : 1 and 1 : 2, the lower DS might be attributed to the insufficient acetic anhydride added, resulting in a lower acetylation reaction. Further, when the NFC : AA ratio was 1 : 1, only the C2 hydroxyl groups were replaced by acetyl groups, as shown in Fig. S1 (see detail in the ESI†). From Fig. 4, with increasing NFC : AA ratio from 1 : 3 to 1 : 6, the increase in DS was large, as a result of C2 and C6 being replaced by acetyl groups, combining with the ^1^H-NMR spectra. These results were similar to the findings of Ifuku *et al.*,^17^ in which the NFC : AA ratio was 1 : 3 and 1 : 5, and the DS was also approximately 0.25 and 1, respectively. At the same time, the dispersion of NFC in aqueous solution was also improved greatly when the ratio of NFC : AA was increased to 1 : 6. Furthermore, the DS was increased dramatically as the NFC : AA ratio was more than 1 : 6, which was mainly attributed to the replacement of the hydroxyl groups on C2, C6, and C3 by acetyl groups, and further improvement in NFC dispersion in aqueous solution. However, the color of NFC was turned to yellow and gradually deepened to brown yellow as the NFC : AA ratio reached more than 1 : 6. One of main reasons for this was the destruction of the cellulose crystalline structure and the internal crystal structure, and the rapid increase in the reaction of cellulose oxidation, dehydration, and condensation.^32^ The change in color would be bound to affect the light transmittance of the NFC films. In addition, as the NFC : AA ratio reached more than 1 : 6, the acetylated NFC was not able to form a film by vacuum filtration on account of the acetylated NFC losing the ability of the natural fibers to create three-dimensional network structures. The main reason for this was the fact that most of the hydroxyl groups on C3, C2, and C6 of the glucose units were replaced by acetyl groups, leading to a rapid increase in hydrophobic groups in NFC and a dramatic decrease in hydrogen bonds. Thus, it was considered that the properties of nanocellulose fibers would be more favorable when the ratio of NFC : AA was less than 1 : 7.

### Effects of acetylation on the thermal properties of NFC films

The thermal performance of NFC films as FOLED substrates is very critical. This is because the manufacturing temperature of many electronic facilities is designed to be ∼150 to 200 °C in order to obtain optimal performance.^33^ The NFC films were characterized by TGA and derivative thermogravimetric (DTG) in order to obtain the information about their thermal behavior, as shown in Fig. S2 (see detail in the ESI†). The TGA and DTG curves of the NFC films produced by different NFC : AA ratios of acetylated NFC were similar to those of NFC. For all samples, a small weight loss as a result of water evaporation was observed at low temperatures (<110 °C). Also, the NFC films showed a typical single-step thermal degradation, which indicated that the thermal degradation of the NFC films were similar to that of cellulose, mainly for the pyrolysis of cellulose.^33^ As previously reported, the degradation of the NFC films mainly occurred between ∼200 to 300 °C according to the DTG analysis.^34^ It can be concluded that the effects of acetylation on the thermal degradation characteristics of NFC films were very small when the modification ratio of NFC : AA was ∼1 : 1–1 : 6.

A high thermal stability of the NFC film is one of advantages of the FOLED substrate. As shown in Table 1, all NFC films had a very low CTE value, and the values were in the range of ∼5.43 to 20.13 ppm K^−1^, compared with plastic substrates (∼20 to 100 ppm K^−1^).^35^ The results illustrated that the NFC films had a predominantly very low CTE value. When the ratio of NFC : AA increased from 1 : 0 to 1 : 3, the CTE of the NFC films decreased from 15.05 ppm K^−1^ to 5.43 ppm K^−1^; a decrease of nearly 64%. One of main reasons was that the surface and amorphous region of cellulose preferentially reacted with the acetyl groups, and a certain number of acetyl groups was introduced to increase the stability of cellulose, causing a limited thermal expansion. However, with the increase of NFC : AA ratio from 1 : 3 to 1 : 6, the CTE value of the NFC films increased to a maximum of 20.13, which was due to a decrease in hydroxyl groups on the NFC, resulting in the interaction force between fibers becoming weakened. A similar study by Yagyu *et al.*^36^ reported that the CTE values of acetylated cellulose nanopaper was maintained at 8.0–11.1 ppm K^−1^, with DS values from 0 to 1.3. Our study indicated that when the DS was approximately 0.24, the acetylated NFC film had a low CTE value of 5.43 ppm K^−1^, which has great significance to the thermal stability of the FOLED substrate. The above conclusions demonstrated that it was very beneficial to decrease the CTE and improve the thermal stability of the NFC films when the ratio of NFC : AA was 1 : 3.

**Table 1:** Effects of different NFC : AA ratios on the CTE of NFC films

NFC : AA ratio	CTE/ppm k^−1^
1 : 0	15.05 ± 0.68
1 : 1	10.20 ± 0.48
1 : 2	8.76 ± 0.50
1 : 3	5.43 ± 0.35
1 : 4	12.57 ± 0.58
1 : 5	18.13 ± 1.12
1 : 6	20.13 ± 1.10

### Effects of acetylation on the optical properties of NFC films

**Fig. 5:**
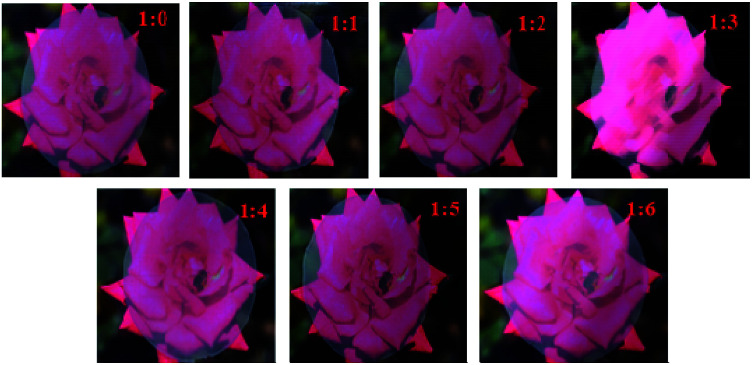
Effects of different NFC : AA ratios on the transparency of NFC films.

Photographs of the NFC films (with a thickness of approximately 42 μm) were taken in a well-lit laboratory to observe their appearance and transparency. The photographs in Fig. 5 showed that that the bees on the rose were clearly visible through the NFC films when the films were placed on background images of a red rose, revealing that all of the NFC films produced by modified NFC with different NFC : AA ratios (1 : 0–1 : 6) had relatively high transparency. Also, the NFC films were observed to be smooth, flat, and uniform.

**Fig. 6:**
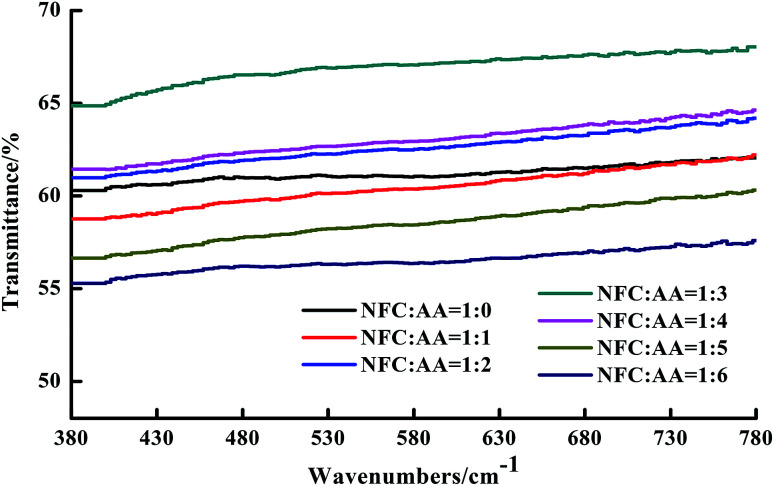
Effects of different NFC : AA ratios on the light transmittance of NFC films.

In order to further determine the transparency of the NFC films, the transmittance of the NFC films in visible light (∼380 to 780 nm) was obtained and is shown in Fig. 6. The light transmittance of the NFC films under the same NFC : AA modification ratios remained basically unchanged, although it was affected to a relatively large degree by different NFC : AA modification ratios. When the ratio of NFC : AA was enhanced from 1 : 0 to 1 : 3, the light transmittance of the NFC films was gradually improved, increasing from 61.05% to 67.16%. However, the light transmittance of the NFC films fell to 56.42% when the ratio of NFC : AA was increased to 1 : 6, which means that the transparency of the NFC film gradually declined with increasing amount of AA. Ifuku *et al.* also reported similar results when he explored the effects of acetylation on the transparency of bacterial cellulose films.^17^ The reason for the deterioration of transparency was mainly attributed to weakening of the bonding force between fibers due to the increase in hydrophobic groups and the reduction in hydroxyl groups with increasing degree of acetylation, resulting in an increase in the porosity of the NFC films.^17^ The above results ensured that the transparency of the NFC films was relatively high and reached a maximum of 65% when the ratio of NFC : AA was 1 : 3.

### Effects of acetylation on mechanical properties of NFC films

**Fig. 7:**
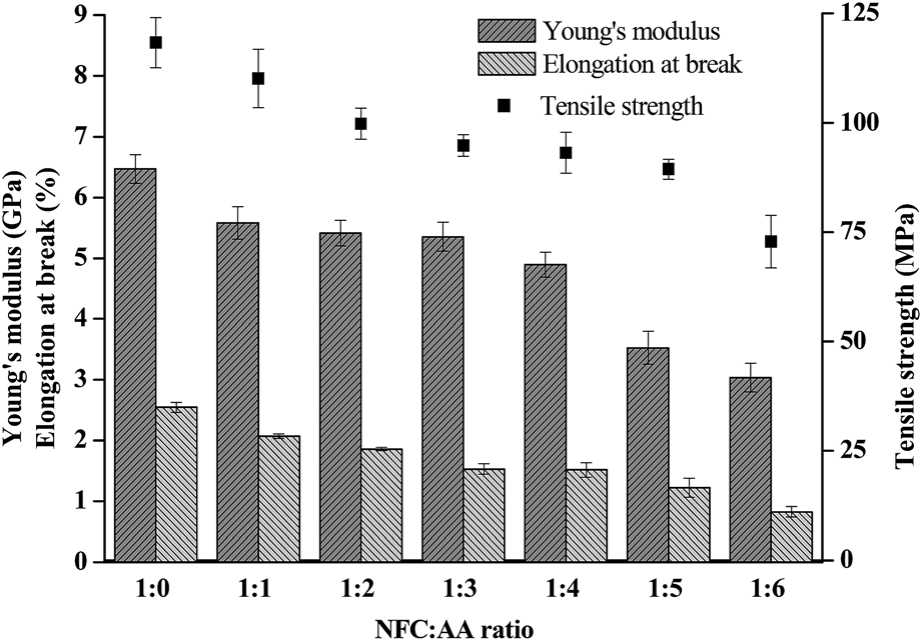
Effects of different NFC : AA ratios on the mechanical properties of NFC films.

Mechanical characteristics is an important index with which to measure the performance of NFC films, and is also an important property of FOLED substrates. In this study, the Young's modulus, tensile strength, and elongation at breakage of the film materials are shown in Fig. 7. The bending degree of the acetylated NFC film is shown in Fig. S3 (see detail in the ESI†). It was reported that the flexibility of film materials was affected by their Young's modulus and elongation at breakage; the lower the Young's modulus and the elongation at breakage, the worse the flexibility of the film materials.^37^ In Fig. 7, as the ratio of NFC : AA increased to 1 : 6, the tensile strength of the NFC films decreased from 118.25 MPa to 72.77 MPa, the Young's modulus reduced from 6.47 GPa to 3.04 GPa, and the elongation at breakage decreased from 2.55% to 0.82%, reduced by 38.46%, 53.04% and 67.84%, respectively. The experimental results showed that different NFC : AA ratios had a considerable influence on the mechanical properties and flexibility of the NFC films. Furthermore, the performance of NFC films, including Young's modulus, tensile strength, and elongation at breakage, suffered a relatively large detriment when the NFC : AA ratio was greater than 1 : 4, as a result of the hydroxyl groups on NFC being gradually substituted with acetyl groups. However, the flexibility of the acetylated NFC films was still excellent (only the images of 1 : 3 NFC : AA ratio are exhibited in Fig. S3† as a representative example).

## Conclusions

NFC produced by mechanical grinding and high-pressure homogenization treatment had an excellent aspect ratio. A smooth NFC film was rapidly produced by simulating the papermaking process. Acetylation improved the dispersion of NFC in aqueous solution and enhanced the thermal stability and transmittance of NFC film. The mechanical properties of NFC film were relatively undamaged by the process of acetylation. However, highly acetylated NFC could not form a film by vacuum filtration if the NFC : AA ratio was more than 1 : 6. The NFC film prepared with acetylated NFC shows potential as a FOLED substrate.

## Conflicts of interest

The authors declare no competing financial interest.

## Supplementary Material

RA-008-C7RA11134G-s001
