# Supplementary material for: Acetylation improves thermal stability and transmittance in FOLED substrates based on nanocellulose films
Source: RSC Adv. 2018 Jan 17;8(7):3619–25. doi: 10.1039/c7ra11134g (PMC9077702; doi:10.1039/c7ra11134g)
Supplement: RA-008-C7RA11134G-s001 [file RA-008-C7RA11134G-s001.pdf]

Electronic Supplementary Material (ESI) for RSC Advances.
This journal is © The Royal Society of Chemistry 2017

# Electronic supplementary information

## Acetylation improving the thermal stability and transmittance of FOLED substrate based nanocellulose film

Shuang Yang[a,b], Qiuxia Xie[a,b], Xiuyu Liu [a,b], Min Wu [a,b], Shuangfei Wang [a,b], and Xueping Song [a,b] *

a. College of Light Industry and Food Engineering, Guangxi University, Nanning, 530004, PR China

b. Guangxi Key Laboratory of Clean Pulp & Papermaking and Pollution Control, Nanning 530004, PR China

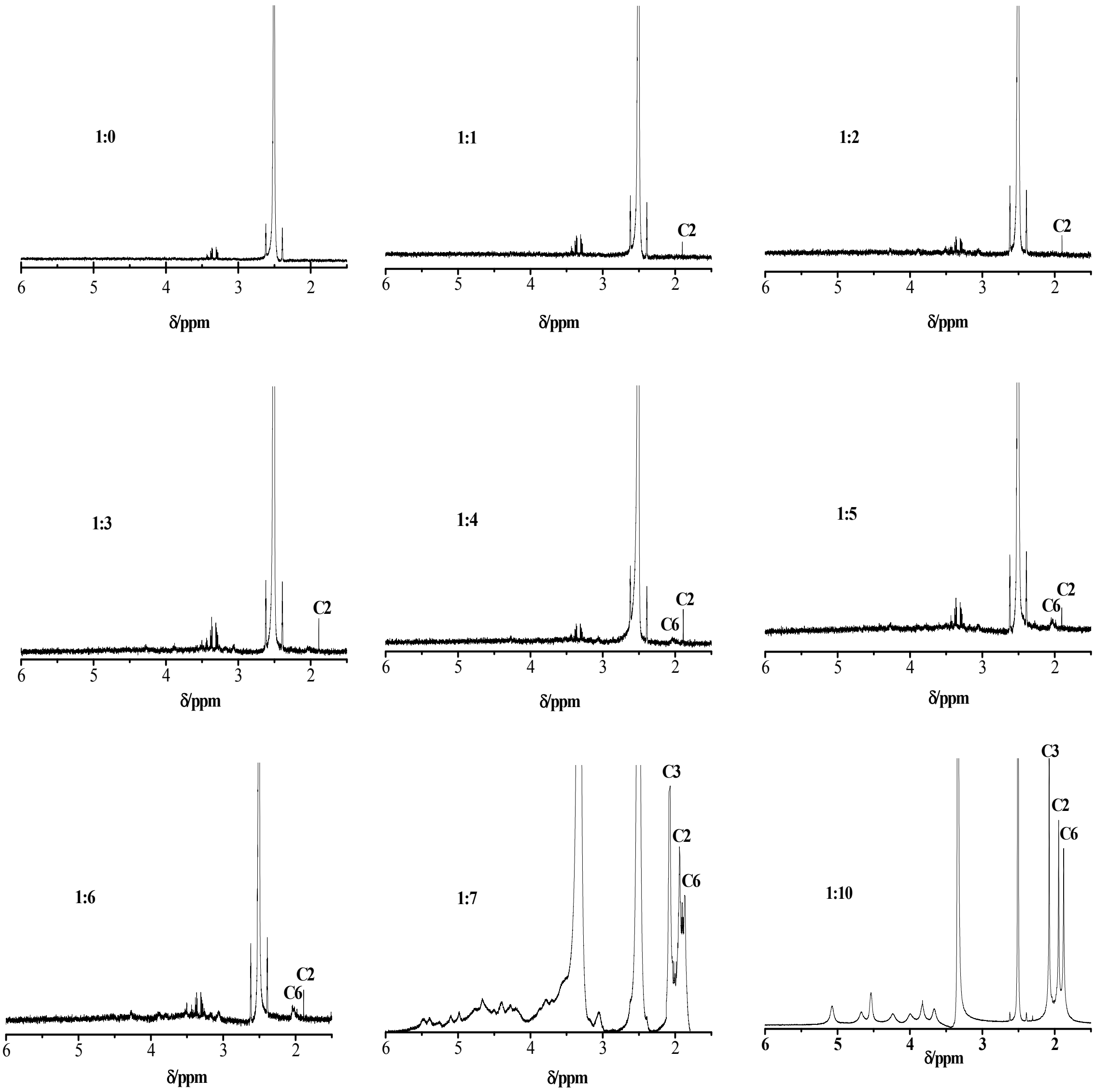


**Fig. S1 Effects of different NFC: AA ratios on the $^1$H-NMR spectra of NFC**

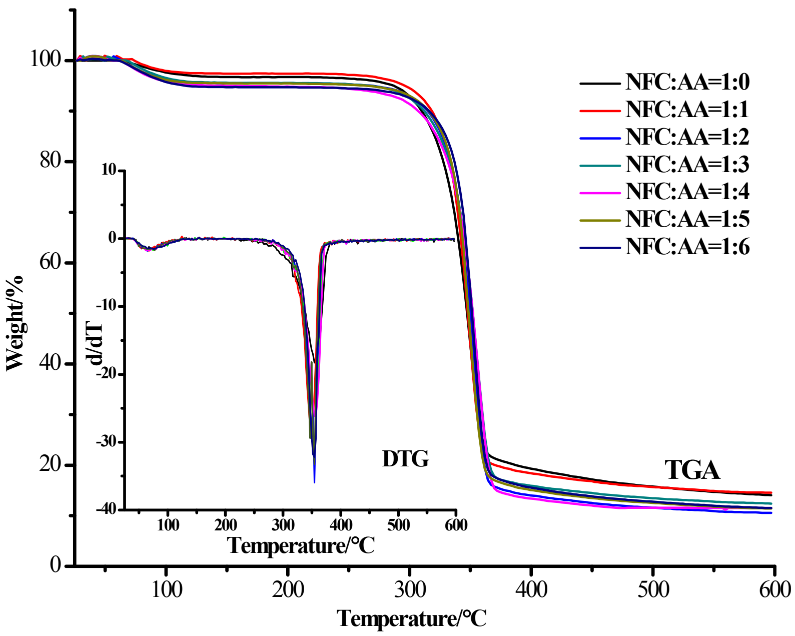


**Fig. S2 Effects of different NFC: AA ratios on the TGA and DTG of NFC films**

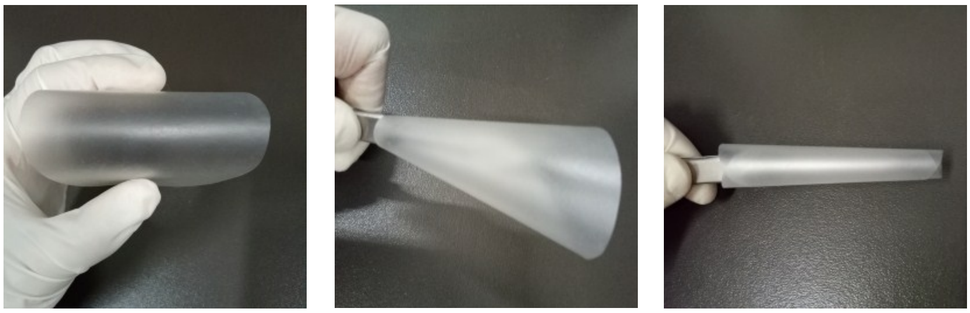

**Fig. S3 Bending degree of acetylated NFC film (NFC: AA ratio was 1:3)**
